# Botulinum toxin in cancer therapy—current perspectives and limitations

**DOI:** 10.1007/s00253-021-11741-w

**Published:** 2021-12-24

**Authors:** Tomasz Grenda, Anna Grenda, Paweł Krawczyk, Krzysztof Kwiatek

**Affiliations:** 1 Department of Hygiene of Animal Feeding Stuffs, National Veterinary Research Institute, Partyzantow Avenue 57, 24-100 Pulawy, Poland; 2 Department of Pneumology, Oncology and Allergology, Medical University of Lublin, Jaczewskiego 8, 20-954 Lublin, Poland

**Keywords:** BoNT; Botulinum toxin, Tumor, Cancer

## Abstract

**Abstract:**

Different serotypes of botulinum toxins (BoNTs) act upon different types of SNARE proteins. This property is used in aesthetic medicine to treat certain eye disorders such as crossed eyes (strabismus) and uncontrolled blinking (blepharospasm), to treat muscle spasms or movement disorders, and, for the two last decades, more and more often, to provide support in cancer therapy, especially so as to obtain analgesic effects upon spastic conditions. The limited literature data also suggests that the addition of BoNTs to the culture of cancer cell lines reduces cell growth, and mitotic activity, and promotes their apoptosis. BoNTs have several advantages that can be emphasized: BoNTs act on both perfusion and oxygenation; moreover, BoNTs are considered to be safe and free of systemic side effects upon administration. Recently, advances in molecular biology techniques have allowed a wide variety of novel BoNT constructs with alternative functions. These constructs could be assessed as potential new classes of anti-cancer drugs. This creates new potential perspectives in the wider use of non-toxic modified BoNT constructs in cancer therapy. In the light of the mentioned premises and existing literature reports, the aim of this review is to summarize current data and reports considering BoNT use in cancer therapy.

***Key points*:**

*•Botulinum toxin (BoNTs) may be useful in cancer treatment.*

*•Botulinum toxin can serve as an analgesic after cancer radiotherapy.*

*•Botulinum toxin has the ability to inhibit tumor growth and promote apoptosis of neoplastic cells.*

## Introduction—mechanism of action of botulinum toxins

Botulinum toxins are produced by the anaerobic spore-forming bacteria of the genus *Clostridium*. The presence of these bacteria has been proven in food products for humans and animals (Maikanov et al, 2019; Grenda et al. 2017). Botulinum toxins are composed of a protoxin complex that is a mixture of proteins containing botulinum neurotoxin (BoNT) and several non-toxic neurotoxin-associated proteins (NAPs). NAPs protect the inherently fragile BoNTs against the hostile environment of the gastrointestinal tract and help BoNTs pass through the intestinal epithelial barrier before they are released into the general circulation. The BoNTs are the most toxic substances naturally occurring in the environment (Gu and Jin 2013). The seven distinct BoNT serotypes are designated by the letters A–G. Among the mentioned serotypes, more than 40 subtypes have been distinguished (Rossetto et al. 2014).

Literature data report that the human nervous system is susceptible to the action of the said A to G BoNT serotypes (Eleopra et al. 2020; Samizadeh and Boulle 2018; Dressler et al. 2019). Moreover, there are chimeric BoNTs that have been described—BoNT/CD and BoNT/DC. These share structures characteristic for both C and D toxin types and the ability to affect the nervous system (Woudstra et al. 2012).

Neurotoxin complexes might differ among particular serotypes with regard to potency binding or the intracellular protein target. BoNT action is based on entering the peripheral cholinergic nerve terminals and inducing cleavage of one up to three core proteins of the SNARE complex (SNAP Receptors, soluble NSF [N-ethylmaleimide-sensitive factor] attachment proteins receptors). The consequence of BoNT action is the blockade of acetylcholine. Generally, paresis is observed 2–5 days after injection and reaches its maximum at 5–6 weeks, lasting for about 2–3 months (Dressler et al. 2005).

The botulinum neurotoxin complex is composed of BoNT molecules with the weight of 150 kDa, as well as non-toxic protein complex that play a protective role against deactivating factors, such as stomach acid. The non-toxic component (neurotoxin-associated proteins, NAPs) consists of hemagglutinin (HA) and non-toxic non-hemagglutinin (NTNH) proteins. The NTNH fraction also includes specific antigenic proteins considered the cause of antibodies formation in BoNT therapy (Jabbari, 2015). The main body of BoNT is composed of a heavy chain (HC, 100 kDa) and a light chain (LC, 50 kDa) connected with a disulfide bridge (Pirazzini et al. 2017).

The action of BoNT is initialized through heavy chain binding (via the receptor binding domain) to polysialogangliosides (PSGs) on the cell surface. Subsequently, the toxin is internalized through binding with another surface receptor. After internalization, the toxin resides within synaptic vesicles. The vesicles are then acidified by the influx of H + ion through vesicular proton pumps, thus activating ACh transporter proteins in the vesicle membrane, which import and concentrate cytosolic ACh within the vesicle. The light chain is translocated to the cytoplasm from inside the vesicles. This is facilitated by the N terminal of the heavy chain (translocation domain). The light chain remains inactive while it remains bound to the rest of the toxin. After translocation, the light chain is released by the action of cleaving enzymes such as heat shock protein 90 (hsp90) and the thioredoxin reductase–thioredoxin system (TrxR-Trx) (Choudhury et al., 2021). The released LC is now active and able to cleave SNARE proteins such as VAMP (vesicle-associated membrane protein), SNAP25 (synaptosomal-associated protein, 25 kDa), and syntaxin (Stx), which are essential for the fusion of vesicles with the presynaptic membrane and for the release of acetylcholine (Pirazzini et al. 2017). The mentioned SNARE proteins play the role of a target for specific toxins. BoNT/A and BoNT/E are responsible for SNAP-25 cleavage, BoNT/B, BoNT/D, BoNT/F, BoNT/G, and BoNT/X – for VAMP cleavage, while BoNT/C is able to cleave SNAP-25 and syntaxin simultaneously. Inactivation of the listed proteins induces blockage of acetylcholine release and temporal and reversible paralysis of muscles (Table 1) (Pirazzini et al. 2017; Car et al. 2021).

**Table 1:** SNARE targets of particular BoNTs

SNARE protein	BoNT type						
	**BoNT/A**	**BoNT/B**	**BoNT/C**	**BoNT/D**	**BoNT/E**	**BoNT/F**	**BoNT/G**
**SNAP-25**	X*	-	X	-	X	-	-
**Syntaxin**	-	-	X	-	-	-	-
**VAMP**	-	X	-	X	-	X	X

^*^X—the cleavage ability of particular SNARE proteins by BoNT

Currently, we are in the era of personalized medicine. The development of molecular diagnostics has led to the point where modern cancer treatment is highly effective with long overall survival (OS) and an improvement in the quality of life compared to chemotherapy, which is highly burdensome for the body and does not always bring satisfactory results. Three major therapeutic strategies are currently used in targeted therapy. The first is the administration of drugs that target specific mutational changes in DNA (genome-targeted therapy), i.e., point mutation, gene fusion/rearrangement in *EGFR* (*Epidermal Growth Factor Receptor*), *ALK* (*ALK Receptor Tyrosine Kinase*), *ROS1* (*ROS Proto-Oncogene 1, Receptor Tyrosine Kinase*), and *NTRK* (*Neurotrophic Receptor Tyrosine Kinase*) genes (Clavé et al., 2019, Grenda et al. 2018; Wu et al. 2021). The second is the use of treatments aimed at blocking the overexpression of tumor cell receptors (brought about by the increased number of gene copies, i.e., *HER2* (*Erb-B2 Receptor Tyrosine Kinase 2*)) (Oh and Bang 2020). The third of the most modern therapeutic strategies is the application of immune checkpoint inhibitors that activate the immune system in such a way that it recognizes “hidden” cancer cells and destroys them (Christofi et al. 2019).

All of the abovementioned treatments contribute to the prolongation and improvement of the cancer patient’s life. Nonetheless, e.g., in England, 45% of all patients diagnosed with cancer had tumor removal surgery as one arm of their primary cancer treatment (National Cancer Registration & Analysis Service and Cancer Research UK). This indicates that surgical intervention and radiation treatment are still very important in the treatment of cancer (Abraha et al. 2018). Therefore, it is important to pay special attention to research on new methods of neo-adjuvant treatment that can reduce the size of the tumor, as well as to develop preparations that can aid in the recovery of patients after tumor removal surgery and prepare them for possible further treatment. This mini-review draws attention to botulinum toxin, which, despite being one of the strongest toxic substances found naturally, when properly prepared, can be used in oncological treatment.

## Botulinum toxin preparations and their clinical use

Nowadays, there are four preparations that are the most commonly used in clinical practice: OnabotulinumtoxinA (ONA; trade name Botox®/Botox® Cosmetic, Vistabel®, Vistabex®), AbobotulinumtoxinA (ABO, trade name: Dysport Therapeutic®), IncobotulinumtoxinA (INCO; trade name: Xeomin®, Bocouture®) and RimabotulinumtoxinB (RIMA, trade name: NeuroBloc®, Myobloc®). Recently, some new formulations of BoNT A have appeared on the market. These include the following: PrabotulinumtoxinA-xvfs (PRA-BoNT/A; trade name: Jeuveau®; Nabota®, Nuceiva®) and daxibotulinumtoxinA (alternative names: DAXI, DAXI-BoNTA; RT002). In addition to the listed preparations, letibotulinumtoxinA (LetiBoNT; trade name: Botulax®) is in phase III clinical trials (Car et al. 2021; Choudhury et al., 2021), while BotulinumtoxinE (alternative name: EB-001), a BoNT type E preparation, is under phase II clinical trials (Yoelin et al., 2018; Choudhury et al., 2021).

All botulinum toxin type A products are derived from subtype A1 organisms. The dissociation of toxin molecule from NAPs occurs at physiological pH (Frevert and Dressler 2010; Eisele et al. 2011). All commercial BoNT preparations contain a vehicle substance (excipient)—human serum albumin (HSA) enhancing the BoNT stability and preventing toxin aggregation or absorption and extending the shelf life (Pickett 2014).

The formats of commercial preparations also differ from each other; e.g., ONA is distributed in vacuum-dried form, while ABO and INCO are freeze-dried. RIMA is distributed in a diluted form and stocked in vials. Before clinical application, ONA, ABO, and INCO should be diluted in saline buffer (Car et al. 2021; Frevert 2015; Matak et al., 2019; Jabbari 2015). Units of various BoNTs are not interchangeable, but sometimes approximation equivalence is used to compare toxin activity, e.g., 1 ONA unit = 1 INCO unit = 2.5 ABO units = 40–50 units of RIMA (Jabbari 2015).

After reconstitution, all the preparations are recommended for immediate use. If not used immediately, in-use storage times and conditions prior to use are the responsibility of the user and would normally not be longer than 24 h at 2° to 8 °C, unless reconstitution/dilution has taken place in controlled and validated aseptic conditions (Botox®, Nuiceva®, Dysport®, Xeomin®). RIMA is recommended for immediate use after dilution (Neuroblock®). However, some studies suggest that ONA can retain efficacy even up to 2 weeks under refrigerated conditions (Yang et al. 2008; Liu et al. 2012). Parsa et al. (2007) showed clinical efficacy after long-term freezing of reconstituted product even up 6 months, which is not normally recommended. The most frequent comparisons of effectiveness among the three most often used BoNT/A preparations (ABO, ONA, and INCO) are described in the literature (De Boulle et al. 2010; Carruthers and Carruthers 2005; Bonaparte et al. 2016; Ferrari et al. 2018). De Boulle et al. (2010) suggest that ABO and ONA have non-parallel dose response curves and their relative potencies differ from each other.

Literature evidence shows that BoNT preparations (ONA, INCO, ABO, and RIMA) have been used for the treatment of cancer pain, post-radiation cancer pain, post-surgical cancer pain, sialorrhea and gustatory sweating, and to evaluate effect on cancer cells and apoptosis (Mittal and Jabbari 2020). Predominantly, the use of ONA was described in clinical trials (Daele et al., 2002; Mittal et al. 2012; Bach et al. 2012; Wittekindt et al. 2006; Layeeque et al. 2004; De Groef et al. 2018; Steffen et al. 2014; Laskawi et al. 2013). The employment of RIMA in cancer therapy has been described only by Cantarella et al. (2010).

These preparations were described in the context of the BoNT effect on local pain after radiation or/and surgery for head and neck or breast cancers. BoNT preparations were also administered in treating clinical problems after parotidectomy, such as fistula, gustatory sweating, gustatory hyperhidrosis, functional hypersalivation, sialorrhea, and sialocele. According to some studies, BoNT preparations act on both perfusion and oxygenation. Moreover, they briefly open tumor vessels, providing an opportunity for more effective destruction of cancer cells by radiotherapy and chemotherapy (hence, increasing the efficacy of both) (Ansiaux et al. 2006; Ansiaux and Gallez 2007).

The use of BoNT preparations has been reported in the context of in vivo and in vitro effects on tumors (Vezdrevanis 2011; He et al. 2016; Coarfa et al 2018; Cheng et al. 2013) and cancer cell lines. Exposure of LNCaP and PC-3 cell lines to ONA treatment (Karsenty et al. 2009), prostate cancer cell lines to IncoA (Proietti et al. 2012), and studies of the effect of ONA on cancer cells implanted into rodent prostates have been performed (Coarfa et al. 2018).

In addition to the aforementioned preparations, experimental studies were conducted on BoNT/C effect on cell lines and malignant tumors in animals. For example, Ulloa et al. (2015) described the treatment of mouse striatum tumors with BoNT/C1, while Rust et al. (2016) conducted experiments on the addition of BoNT/C to human neuroblastoma cell cultures. Preparations based on BoNT/C are not yet approved, but the mentioned studies (conducted in vitro) indicate the effectiveness of this substance in tumor cell apoptosis stimulation (Ulloa et al. 2015; Rust et al. 2016).

## Analgesic effect of botulinum toxins in cancer treatment

It is estimated that about 25% of all oncology patients who have undergone surgery or radiation therapy experience postsurgical or post-radiation pain (Jabbari, 2015; Kehlet et al. 2006). Pain manifestation is observed after surgeries such as the following: limb amputation, tracheotomy, mastectomy, pneumonectomy and dissection of axillary, cervical or inguinal lymph nodes (Jabbari, 2015; Mittal and Jabbari 2020). Medications used the most often in the case of moderate to severe postsurgical pain are based on opioids. These come with extreme abuse potential. Moreover, this therapy is not always effective in some cancer pain (Mittal and Jabbari 2020). Indeed, up to 30% of all patients after radiotherapy for head and neck cancer related to the development of fibrosis, scar, and keloid formation suffer from pain not amenable to standard treatment (Mittal and Jabbari 2020).

Complex studies can be found in current literature that report on the results of oncology patient pharmacological use of botulinum toxins that include evaluation of treatment effect using different types of pain scales, such as VAS (Visual Analogue Scale), FDSNP (Functional Disability Scale for Neck Pain), and PGIC (Patient Global Impression of Change) (Rostami et al. 2016; Mailly et al. 2019). Analgesic effect was studied in regard to head and neck and breast cancer and after or during radiation, surgery, or chemotherapy (Ferrari et al. 2018; Mittal and Jabbari 2020).

After radiotherapy, peripheral nerves and muscles are affected and the manifestation of damage appears in acute or late course. The late effects are generally observed more often, and their manifestation is considered to be dose-dependent (Gillette et al. 1995). Experiments conducted on animals show histologic changes in muscle appearance after 3 or 4 weeks post-radiotherapy, and muscle degeneration and focal areas of capillary loss develop 2 to 4 months after radiation therapy at a dose of 20 Gy. A decrease in proteoglycans in the extracellular matrix, as well as an increase in collagen leading to tissue fibrosis, was observed.

The negative effect of radiotherapy could progress for up to 2 or even 5 years post-treatment and it is correlated with radiotherapy dose (Van Daele et al. 2002). Treatment of these problems based on analgesics and administration of trolamine, *Calendula officinalis*, or hyaluronic acid application is effective only temporarily and is not sustainable (Park and Park 2017; Safarpour and Jabbari 2018; Shaw et al. 2019). BoNT application seems to be more effective, has a relatively low side-effect profile, and has low risk of drug interactions (Mittal et al. 2012).

Van Daele et al. provide one of the earliest observations of analgesic effect following BoNT injections (Van Daele et al. 2002). In a preliminary study, they reported the application of ONA in doses of 20–25 units on six oncological patients after radiation and chemotherapy of head and neck carcinoma. In this study, pain was estimated by VAS. Patients’ complaints ranged from nondescript requests for pain medication for “neck muscle pain,” to more specific descriptions of spasms occurring in the sternocleidomastoid muscle lasting seconds to minutes. Based on the obtained results, one patient was classified as a non-responder. The EMG (electromyography) activity of the mentioned patient was evaluated as very low after toxin injection. A second patient had an inadequate response after injections. The remaining four patients obtained complete relief of pain after 2 injections an average of 3–4 months apart. This study had a preliminary character and it only described observations based on individual patient perception after BoNT injection without control (lack of a placebo group).

In addition, Mittal et al. (2012) reported upon the relief of refractory post-radiation or postsurgical cancer pain after local treatment with ONA (Mittal et al. 2012). The authors described the effect of ONA in seven cancer patients who suffered from severe focal pain (as estimated by VAS) at the site of local surgery or radiotherapy, or both. In the study, ONA (20–100 units) was injected into the focal pain areas (skin or muscle or both). Five of seven patients were then followed beyond 1 year (1.5–5 years) and received a retreatment with ONA. The authors observed response on the patient global assessment as satisfactory (two patients) or very satisfactory (five patients). They concluded that local treatment with ONA can significantly reduce pain and improve life quality of cancer patients suffering from pain in areas affected by surgical intervention and radiation therapy (Mittal et al. 2012). Of note, the described BoNT therapy was well tolerated in cancer patients. This report was based, however, only on VAS results obtained from patients after BoNT application, without a placebo group.

Wittekindt et al. (2006) described a clinical trial (without a placebo group) on 23 patients with pain after surgery and radiation in the neck area. The patients had previously undergone extensive conservative treatment for neck and shoulder pain. Patients were divided into a low-dose (*n* = 13) (ONA, 80–120 U) and a high-dose group (*n* = 13) (ONA 160–240 U). Pain and quality of life were assessed at day 0 and day 28. Patients in the low-dose group showed an improvement in quality of life and a significant pain reduction as evaluated by VAS (*p* < 0.05). In contrast, the mean pain VAS values in the high-dose group did not improve significantly. No serious side-effects were observed. This study drew attention to the possible lack of high dose effectiveness of OnaA injections in pain relief. However, the results need verification in clinical trials involving a placebo group.

Rostami et al. (2016) described the application of INCO to twelve patients who had surgery or radiation for treatment of head and neck cancer or breast cancer and for whom at least two analgesic medications for pain control failed. Patients were prospectively enrolled to the study and were injected with up to 100 units of INCO intramuscularly or subcutaneously depending on the type and location of pain (muscle cramp or neuropathic pain). Two patients died during the observation, one was excluded from the study due to a skin reaction and another one—because of poor general condition. The remaining patients showed significant improvement as measured by VAS, and reported significant satisfaction as self-assessed using the Patients’ Global Impression of Change scale (7 out of 8 patients reported a reduction in pain intensity). Furthermore, three of the 8 patients reported significant improvement in quality of life. This study reported evaluation of pain by two scales, however, without a control group (placebo).

Another study without a placebo group was published by Mailly et al. (2019). They described the application of IncoA and ABO in patients undergoing radiation and surgery for head and neck cancer. Pain therein was evaluated by each of 16 patients on a visual analogue scale before, and 1 month after the injection. The authors noted that the differences in perception of pain were statistically significant (*p* < 0.01). Major response occurred in 15 patients and complete response in 11 patients.

The most complex studies on BoNT injection influence on pain reduction were conducted by Layeeque et al. (2004) and De Groef et al. (2018). Layeeque et al. (2004) described the results of a study conducted on 48 patients who had undergone complete reconstruction with permanent implant placement, as well as postsubpectoral tissue expansion following mastectomy and immediate insertion of tissue expander. Here, 22 (46%) were administered ONA (intervention group) and 26 (54%) were not (control group). The intervention group experienced a significant improvement of pain intensity (*p* < 0.0001), and there were no BoNT-related complications. The authors concluded that muscular infiltration of botulinum toxin in patients undergoing mastectomy and tissue expander placement significantly reduced postoperative pain and discomfort without complications.

In turn, De Groef et al. (2018) investigated the effect of a single ONA injection after breast cancer surgery. The authors designed a double-blinded, randomized, placebo-controlled (saline injection) trial. Measures were taken before the intervention and at 1, 3, and 6 months follow-up. The authors evaluated changes in VAS. They noted no significant changes in pain intensity after 3 months; however, after 6 months follow-up, a significant change in upper limb pain intensity was reported between the groups in favor of the intervention group. The authors concluded that a single BoNT application in combination with an individual physical therapy program significantly decreased pain intensity within the upper limb in breast cancer survivors for up to 6 months.

## In vitro and in vivo effectiveness of BoNT application to cancer cell lines and malignant tumors

In the last two decades, many studies considering the potential application of BoNTs for reduction of tumor size or for the triggering of cancer cells apoptosis have appeared. These studies were conducted using either animal or human models or in vitro carried out with direct application of BoNT’s into tumor cell lines. The mentioned in vitro studies were undertaken using various cell lines derived from tumors such as neuroblastoma, endocrine tumor, breast cancer, prostate cancer, colorectal cancer, or pancreatic cancer cells. One of the earliest studies was that by Huang et al. (1998). This experiment was performed using insulin-secreting HIT-T15 cells. The authors demonstrated that insulin secretion could be regulated by transient transfection of BoNT/A into SNAP-25. The obtained results are promising for BoNT/A use in endocrine tumor treatment.

Another experiment was conducted by Karsenty et al. (2009). They evaluated influence of ONA treatment on the proliferation of PC‐3 and LNCaP cell lines (prostate cancer). The experiment included non-exposure to BoNT as control. The authors observed that BoNT/A significantly reduced LNCaP cell proliferation and increased apoptosis in a dose‐dependent manner, but did not affect PC‐3. The SV2 (synaptic vesicle glycoprotein 2, which is the target of this neurotoxin) receptor was present in both cell lines at a ratio of 4:1 (LNCaP/PC‐3). They estimated that 1 unit of ONA significantly influenced growth rate (lower) and PSA (Prostate Specific Antigen) progression (slower) over 28 days, in comparison to controls. The authors emphasized that after ONA application, there were significantly more apoptotic cells as compared to controls.

The influence on prostate cancer cell proliferation was also investigated by Proietti et al. (2012). They subjected the prostate cell lines LNCap and PC-3 to different IncoA doses, and noticed a 20% reduction of cell growth in LNCaP and 25% in PC-3 after 96 h of INCO administration. In this research, they observed SV2 receptor expression in both experimented cell lines. Herein, cPLA2-α (Cytosolic Phospholipase A2-α) expression was not observed in LnCaP; however, in PC-3, a high expression of cPLA2-α was observed which was not modified after IncoA treatment. The authors concluded that in both LNCap and PC-3 cell lines, the expression of P-Ser505-cPLA2-α (phosphorylated enzyme) increased significantly after treatment with IncoA [10 U/ml].

In turn, the influence of BoNT/A treatment on breast cell lines was investigated by Bandala et al. in two studies from 2013 (Bandala et al. 2013) and 2015 (Bandala et al. 2015). In the study conducted in 2013, they evaluated different doses ONA influence on T47D cell line apoptosis. The authors observed that BoNT/A exerted greater cytotoxic activity in T47D cells, in comparison to that with MCF10A normal cells. They concluded that botulinum toxin A induced caspase-3 and -7 dependent apoptotic processes in the T47D breast cancer cell line.

In 2015, Bandala et al. (2015) published the results of the influence of ONA application on the presence of the SV2 receptor in three breast cancer cell lines: T47D, MDA-MB-231, and MDA-MB-453. They observed that in all three cancer cell lines, botulinum diminished SV2 receptor expression. The authors concluded that SV2 could be a molecular marker in breast cancer and its expression could be regulated by BoNT/A. These findings suggested possible utilization of BoNT with trastusumab conjugate in breast cancer therapy.

Beyond the aforementioned, Hajighasemlou et al. (2015) described the impact of BoNT/A on two breast cancer cell lines: SK-BR-3 and BT-474. They noted that Herceptin-BoNT/A bioconjugate significantly improved Herceptin efficacy in both breast cancer cell lines when compared to the control group (BoNT and trastuzumab used separately). The authors concluded that Toxin-Herceptin bioconjugation could be a potential candidate for treating breast cancer patients with HER2 receptor overexpression (Hajighasemlou et al. 2015).

The results of experiments that used neuroblastoma cell lines are also interesting. Such research was described by Rust et al. (2016). The authors investigated the influence of BoNT/C proteolytic activity on human neuroblastoma cell lines: SiMa and SH-SY5Y (BoNT/C is usually considered one of the main etiological factors of animal botulism, while neuroblastomas constitute a major cause of cancer-related deaths in young children). They saw that human neuroblastoma cells: SiMa and SH-SY5Y acquired a neuronal phenotype that was evidenced by axonal growth and expression of neuronal markers. BoNT/C, which cleaves neuron-specific SNAP25 and syntaxin1, caused apoptotic death only in differentiated neuroblastoma cells.

Schebl (2019) undertook a complex study on the antitumor potency of ONA and captopril. In this, the cytotoxic effect of captopril and BoNT-A was determined using MTT assay against colon (HCT116) and prostate cancer (DU145) cell lines, and was compared to their effect on normal Vero cell lines. The results revealed that both drugs used in the experiment had significant inhibitory potential on cellular proliferation and the ability of cancer cells to migrate in scratched monolayers. This effect was obviously inhibited, along with a decrease in their concentrations. In the experiment, TP53 (Tumor Protein 53) expression levels in DU145 cells treated with captopril and ONA were elevated 4 and 2.5 times in regard to control, respectively. However, a lower level of apoptosis induction in HCT116 cells was observed. The authors concluded that BoNT-A and captopril could present potential anti-cancer activity through triggering cancer cells towards self-destruction.

Besides the abovementioned in vitro tests, in vivo experiments were conducted on animals (mice model) or directly by application of BoNTs into human tumors. Ansiaux et al. (2006) conducted an experiment using NMRI mice that involved implantation of two tumor models in the thigh of mice: a Syngeneic FSa II fibrosarcoma model in C3H/HeOuJIco mice and a transplantable mouse liver tumor model.

The experiments were carried out with ONA local injections into mouse tumors (fibrosarcoma FSaII, hepatocarcinoma transplantable liver tumor). Oxygenation of tumors was measured by using electron paramagnetic resonance oximetry in vivo. Perfusion of tumors was measured also in vivo by using contrast-enhanced magnetic resonance imaging. The isolated arteries were mounted in a wire myograph to monitor specifically the neurogenic tone developed by arterioles that were co-opted by the surrounding growing tumor cells. The authors showed that local administration of ONA (two sites; dose, 29 units/kg) significantly increased tumor oxygenation and perfusion, leading to a substantial improvement in the tumor response to radiotherapy (20 Gy of 250-kV radiation) and chemotherapy (cyclophosphamide, 50 mg/kg). The experiment demonstrated that ONA could inhibit the neurogenic tone in the tumor vasculature (Ansiaux, 2006).

Vezdrevanis (2011) presented the case report of a patient with metastatic prostate cancer (PCa). The patient underwent castration-refractory prostate cancer (CRPC) and additionally was treated with dexamethasone and lanreotide, as well as alfuzosin. The patient had an intraprostatic injection of ABO in a dose of 1000 units diluted in 0.5% adrenaline solution in order to relieve his prostate obstruction. Immediately after ABO injection, alfuzosin was discontinued and the patient received a 2-week cycle of capecitabine. Moreover, Finasteride was added to his treatment. After 1 month, the ultrasonographic examination showed 30% reduction in tumor size.

Another experiment in vivo was described by Ulloa et al. (2015). The authors examined the influence of BoNT/C1 on the glioblastoma (GBM is the most prevalent adult brain tumor and comes with a median overall survival of 15 months from diagnosis despite the treatment applied). In the work, U373 cells were pre-treated with BoNT/C1 and then injected into the brain of immuno-compromised mice (striatum of the right brain hemisphere). The authors observed that the blockade of the SNARE protein and Syntaxin 1 function impaired GBM cell proliferation. They noticed that Stx1 has lost their function in GBM cells, and botulinum toxins brought about a reduction of GBM growth after U373 cell grafting.

He et al. (2016) described another in vivo test on mice with pancreatic cancer treated with OnaA. The mice from the experimental group were injected with MIA PaCa-2 cells pre-treated with ONA. Tumor size and apoptotic count were measured. The authors noted that tumor size was decreased and apoptotic rate increased in animals injected with PaCA treated with ONA, in comparison to the control group. They concluded that the neural microenvironment may play an important role in the progression of PaCA. This discovery could lead to the development of novel, nerve-targeted adjuvant therapies for this cancer.

Coarfa et al. (2018) conducted a complex study on animals and a clinical trial on humans with prostate cancer. The authors denervated rodent prostates (rats and mice) using ONA, before orthotopic implantation of cancer cells VCaP. In addition, they performed a clinical trial with ONA as neoadjuvant treatment in prostate cancer patients before prostatectomy. In the first experiment, the authors observed a reduction of tumor incidence and tumor size in mice. In the other, treatment of patients with prostate cancer using ONA resulted in increased apoptosis of cancer cells. Coarfa et al. identified a similar profile of gene expression responsible for denervation in tumors arising in denervated rodent prostates. The authors concluded that nerves play a role in the homeostasis of normal epithelial tissues and they are involved in prostate cancer cell survival.

In contrast, Cheng et al. (2013) described an experiment on mice injected with prostate cancer cell lines (LNCaP and PC3) pre-treated with ONA. In the study, the same cell lines were incubated with ONA in cell cultures. They concluded that ONA did not affect the growth of LNCaP or PC3 cells in vitro and in vivo or produce significant anti‐tumor effects.

## Limitations and future perspectives of BoNT application in cancer therapy

The use of BoNT in cancer therapy carries a risk of potential adverse effect occurrence. Theoretically, it is possible to bring about sustained systemic botulism after injection that could spread beyond the site of injection. However, potential benefits from BoNT “central effects” blockade have been evidenced. Some patients experience disproportionate muscle weakness or clinical benefit for many months, exceeding the average duration of peripheral chemo-denervation. Moreover, injections may improve muscle tone and function in non-treated body parts. Overall, BoNT-related central effects and consecutive modulation and/or reorganization of the brain may not solely be considered “side-effects,” but rather an additional therapeutic impact responsible for a number of clinical observations that cannot be explained by merely peripheral actions (Weise et al., 2019; Hallet 2018).

It should be underlined that patients must be thoroughly selected to any clinical trials. Contraindications in BoNTs use in cancer therapy are pregnancy or lactation. Neurological diseases that could lead to neuromuscular crisis should also be taken into account as excluding factors, e.g., Lambert-Eaton syndrome. Moreover, toxin therapy should not be provided to patients treated with concomitant aminoglycoside antibiotics, like gentamicin, kanamycin, streptomycin, dihydrostreptomycin, neomycin, netilmicin, spectonomycin, or kanamycin. The listed medicines are considered to be able to interfere with BoNTs and prolong their neurotoxic action. Beyond the previous, BoNT therapy should be avoided in patients receiving one of the following medications that could interfere with BoNTs or cause an infection: tetracyclines, linkomycin, polymyxin, chloroquine, calcium channel antagonists, penicillamine, local anesthetics (e.g., lidocaine), cyclosporine, quinine (Pero et al. 2018, Wollina and Konrad, 2005). In addition, some patient conditions are risk factors of infectious disease occurrence after BoNT injection. Appropriate precautions should be taken when patients suffer from diabetes, polymyositis, alcoholism, and immunocompromising conditions (Pero et al. 2018).

BoNT preparations could potentially affect the function of the cardiovascular system. Claus et al. (1995) described significant changes of selected heart rate parameters after application of ABO to 26 patients with torticollis. Despite the lack of clinical symptoms, arrhythmias or any other remote adverse effects being observed, the ABO had a noticeable influence on the attenuation of the mentioned parameters.

Moreover, immunoresistance to BoNT is pointed out as a possible noresponsiveness effect. This is considered to be induced by the development of neutralizing antibodies against the toxin. The formation of neutralizing antibodies is increased by high BoNT doses, also by a short time period between injections. It is believed that sharp increases in toxin dose within less than 1 month could be dangerous and cause lack of response to BoNT therapy (Currà and Berardelli 2009; Benecke 2012). However, there are many reasons why initial responders lose their response. This could be due to development of neutralizing antibodies (immunoresistance). According to a study by Dressler (2004), in the portion of patients with SNR due to neutralizing antibodies, 81% begin initially with a partial loss of effect before progressing to complete loss over an average of 2.5 injections (Dressler, 2004). In a meta-analysis by Fabbri et al., the prevalence of neutralizing antibodies was 3.5% among clinically responding patients and 53.5% in patients with SNR, but half of the patients with SNR did not have neutralizing antibodies (Fabbri et al., 2016). Hence, non-response has remained a complex and unresolved problem, limiting the use of BoNTs in therapies (Bellows and Jankovic, 2019).

Still, the use of BoNTs in cancer therapy continues to steadily expand, and further applications will be developed in the future. Adverse effects have been very rarely indicated during cancer therapy and usually the symptoms were rarely reported and limited only to non-significant paresis in the injected area, dry mouth, and limited hematoma. However, the safe utilization of BoNTs requires knowledge of its indications and pharmacology, as well as the anatomy of the treated muscles to avoid serious complications.

In order to assure safe utilization of BoNTs and effective analgesic or therapeutic effect, new non-toxic recombinants of BoNTs are under consideration for wider application in the future (Fonfria et al. 2018; Whitt et al. 2020).

Whitt et al. (2020) reported the development of a recombinant heavy chain receptor-binding domain (rHCR) of BoNT A which is able to specifically target the synaptic vesicle 2 surface receptor that is abundantly expressed in multiple neuroendocrine tumors. The authors noticed that the expression of neuroendocrine differentiation markers chromogranin A (CgA) and achaete-scute complex 1 (ASCL1) were significantly reduced when cells were treated with rHCR. Here, rHCR was conjugated to the antimitotic agent—monomethyl auristatin E (MMAE). In their experiments, the authors noticed significant suppression of pancreatic cancer and medullary thyroid cancer cell proliferation by rHCR-MMAE. Moreover, no suppression of growth of pulmonary fibroblasts and cortical neuron control cell lines was observed.

Whitt et al. also undertook experiments on mice treated with rHCR-MMAE. Here, in vivo testing indicated that rHCR-MMAE significantly reduced tumor volume in mouse xenografts with no adverse effects. The authors concluded that the obtained results suggest recombinant HCR of BoNT/A preferentially targeted neuroendocrine cancer cells without the neurotoxicity of the full BoNT/A. Moreover, SV2 is a specific and promising target for delivering drugs to neuroendocrine tumors (Whitt et al. 2020).

## Conclusions

Literature data reveal that local injections of botulinum neurotoxins can significantly reduce the local pain experienced by cancer patients after surgery and radiation therapy. The dose of BoNTs is strictly dependent on individual response to treatment, kind of preparations, and severity of symptoms. Adverse effects are rarely reported in literature and limited only to non-significant symptoms. In the last two decades, many studies considering the potential application on BoNTs for the reduction of tumor size or for triggering cancer cell apoptosis have appeared. These studies were conducted in vivo in animal, as well as human models or in vitro in tumor cell lines. The results of the mentioned experiments indicate the possibility of non-invasive and very effective therapeutic use of BoNTs in the therapy of different types of neoplasms. Some doubts are linked with possible toxic and systemic influence of BoNTs on cancer patients. However, knowledge regarding the safe utilization of botulinum toxins has developed and new potentially non-toxic BoNT-constructs have appeared. These new constructs show a wide range of therapeutic effect and could be effectively applied in the near future. Overall, it can be said that the field of application of BoNTs is increasing every year and they have become recognized as new alternative analgesic agents and anti-cancer medication.
